# Proximal humeral fractures in children - controversies in decision making

**DOI:** 10.1007/s00068-024-02534-7

**Published:** 2024-04-30

**Authors:** Philipp Schippers, Erol Gercek, Dorien Schneidmüller, Peter C. Strohm, Christian Ruckes, Erik Wegner, Andreas Baranowski, Sven-Oliver Dietz

**Affiliations:** 1 Department of Orthopedics and Traumatology, University Medical Center of the Johannes Gutenberg University, Langenbeckstr. 1, 55131 Mainz, Germany; 2 Department of Trauma Surgery, BG Unfallklinik Murnau, Murnau, Germany; 3 Clinic of Orthopedics and Trauma Surgery, Sozialstiftung Bamberg, Bamberg, Germany; 4 Interdisciplinary Center for Clinical Trials Mainz, University Medical Center, Johannes Gutenberg University Mainz, 55131 Mainz, Germany

**Keywords:** Proximal humeral fractures in children, Fracture angulation, Consensus

## Abstract

**Background:**

Proximal humeral fractures in children are rare and usually treated non-operatively, especially in children younger than ten. The decision between operative and non-operative treatment is mostly based on age and fracture angulation. In the current literature, diverging recommendations regarding fracture angulation that is still tolerable for non-operative treatment can be found. Besides, there is no consensus on how fracture angulation should be determined. This study aimed to determine whether leading experts in pediatric trauma surgery in Germany showed agreement concerning the measurement of fracture angulation, deciding between operative and non-operative treatment, and choosing a treatment modality.

**Methods:**

Twenty radiographs showing a proximal humeral fracture and the patient’s age were assessed twice by twenty-two senior members of the “Section of Pediatric Traumatology of the German Association for Trauma Surgery”. Experts determined the fracture angulation and chose between several operative and non-operative treatment modalities. The mean of individual standard deviations was calculated to estimate the accuracy of single measurements for fracture angulation. Besides Intra-Class Correlation and Fleiss’ Kappa coefficients were determined.

**Results:**

For fracture angulation, experts showed moderate (ICC = 0.60) interobserver and excellent (ICC = 0.90) intraobserver agreement. For the treatment decision, there was fair (Kappa = 0.38) interobserver and substantial (Kappa = 0.77) intraobserver agreement. Finally, experts preferred ESIN over K-wires for operative and a Gilchrist over a Cuff/Collar for non-operative treatment.

**Conclusions:**

Firstly, there is a need for consensus among experts on how fracture angulation in PHFs in children should be reliably determined. Our data indicate that choosing one method everybody agrees to use could be more helpful than using the most sophisticated. However, the overall importance of fracture angulation should also be critically discussed. Finally, experts should agree on treatment algorithms that could translate into guidelines to standardize the care and perform reliable outcome studies.

**Level of evidence:**

III.

## Introduction

Proximal humeral fractures (PHFs) in children are rare and comprise about 2% of all pediatric fractures [1]. More commonly affecting boys [2], the most frequent etiology is a backward fall on the extended arm [3]. The diagnosis is usually confirmed by radiographs [4].

Due to the high remodeling potential of the proximal humeral physis [5], the majority of fractures can be treated non-operatively, especially in children below ten years [3, 4, 6–12]. In cases of fracture dislocation, soft tissue entrapment, especially of the long head of the biceps tendon, is a feared and sometimes underestimated complication that may need open reduction and internal fixation (ORIF) [13, 14]. In all other cases that require surgery, closed reduction and internal fixation (CRIF) with Elastic Stable Intramedullary Nailing (ESIN) or K-wires is usually possible [15–18].

The Neer-Horowitz (NH) classification of proximal humeral metaphyseal fractures distinguishes four grades based on the displacement in relation to the shaft [19]. For epiphyseal fractures, the Salter-Harris (SH) classification can be used. However, the NH and the SH classifications have shown only fair to good inter- and intraobserver reliability (0.32–0.60) [20]. Apart from age and the NH and SH classifications, fracture angulation is an important impact factor guiding the treatment. Yet, there is no clear consensus on what fracture angulation is still tolerable for non-operative treatment, especially with regard to age [3, 4, 6–11]. Besides, there are diverging propositions on how fracture angulation should be determined. Burke et al. proposed a measurement for PHFs in children that uses a method similar to the epiphyseal-shaft angle for slipped capital femoral epiphysis. Using this new method, they reported excellent inter- and intraobserver reliability in a single-center study using seven observers [20].

This study aimed to evaluate the consensus among experts on determining fracture angulation and the treatment decision.

## Materials and methods

### Population

Expecting Intra-Class Correlation (ICC) coefficients higher than 0.7, an accuracy of 14% was achievable using twenty patients and twenty-two observers. Even when assuming an ICC of 0.65 or 0.60 an accuracy of 16% or 17% was achievable, respectively [21]. Thus, twenty patients below fifteen years who sustained a proximal humeral fracture between 2019 and 2022 were retrieved from our database. Their radiographs were completely anonymized, with only the age (in full years) written on them. The mean age was 9.8 years (± 3.3). Half of the patients were ten years old or younger, and the other half were older than ten years. The population included fifteen females and five males.

### Observers

Twenty-two senior members from the “Section of Pediatric Traumatology of the German Association for Trauma Surgery” (“*Sektion Kinder-Traumatologie*”, SKT der Deutschen Gesellschaft für Unfallchirurgie (DGU)) were asked to participate in the study. All committee members are fellowship-trained trauma surgeons with long-time expertise in pediatric traumatology and work in Level-one trauma centers in Germany.

### Measurements

On each radiograph, observers were asked to determine the fracture angulation. Purposely, there was no obligation to apply a specific measurement technique. Besides, observers were asked to choose an operative (ESIN, K-wire, other) or non-operative (Gilchrist, Cuff/Collar, other) procedure. They could state if more imaging was necessary to make a treatment decision.

### Image analysis

**Fig. 1:**
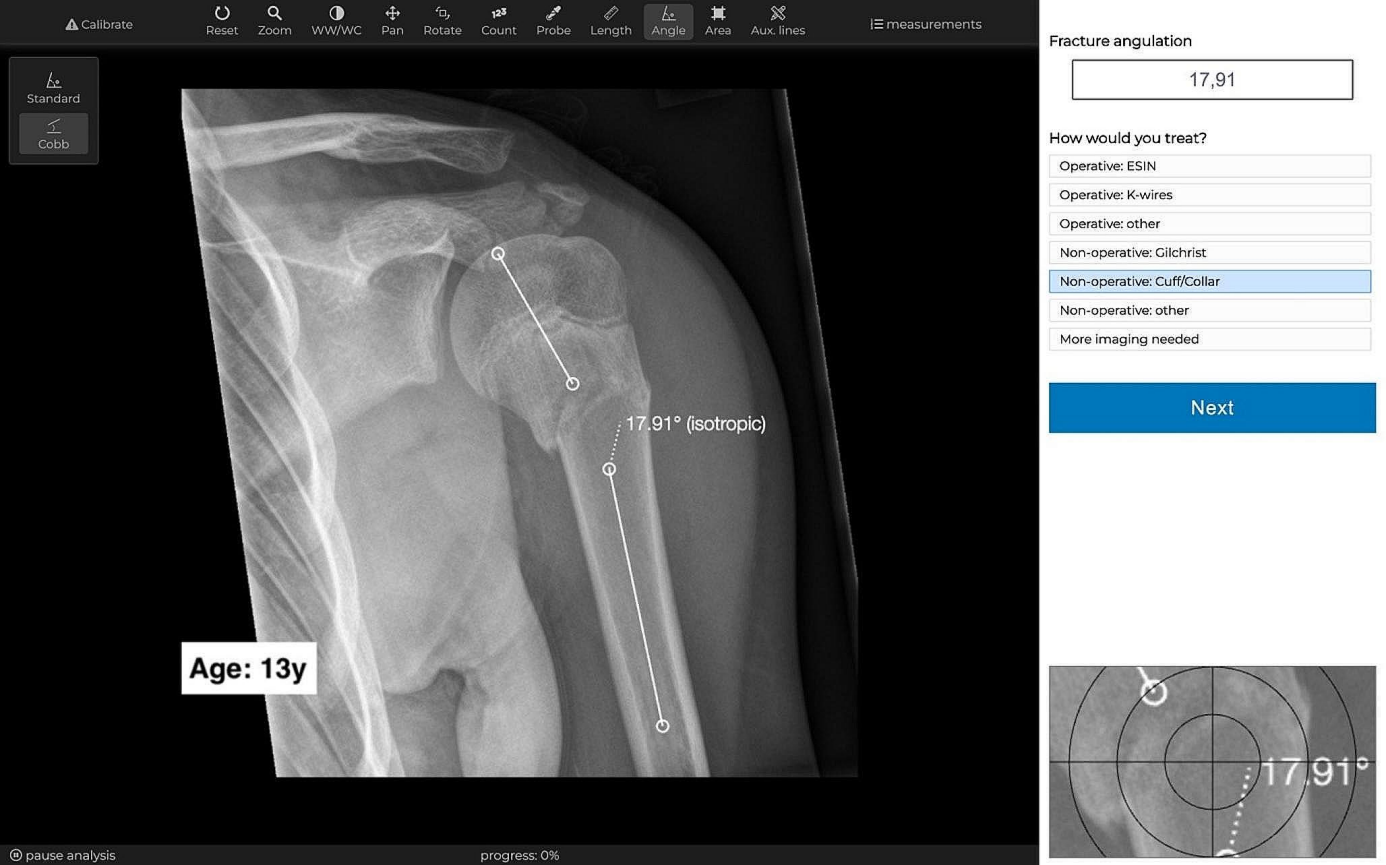
Image analysis was performed blinded and in random order using the online-tool Tyche Twenty-two experts in pediatric traumatology assessed twenty radiographs twice using the online-tool Tyche. Images were shown in random order with standard tools for analysis. On the same window was an input field to store the fracture angulation and a single-choice question with seven answers to choose operative and non-operative treatment. Results were immediately visible to the project manager

The recently introduced online-tool Tyche™ v1.0 (Mainz, Germany) [22–24] was utilized to facilitate a multi-center study including experts from different hospitals. Fully anonymized images were temporarily uploaded in JPEG format to Tyche, where only dedicated observers had temporary access via encrypted connections. Images were analyzed blinded, in random order, and with means to store results online on the same window. Observers could use standard imaging tools like zoom and contrast and the standard and Cobb angle tools (Fig. 1). Results were immediately merged and visible to the project manager.

### Statistical analysis

Mean values with standard deviations (SD) were calculated for the fracture angulation. The accuracy of single measurements was calculated as described by Popović et al. [25]: For every image, the SD between the observers was calculated. Then, the average of these SDs was calculated and termed the “mean of individual standard deviations”. Lower values indicate higher accuracy.

To assess the measurement reliability of the fracture angulation, Intra-Class Correlation (ICC) coefficients were calculated. For interobserver reliability, ICC(3,k) was used; for intraobserver reliability, ICC (3,1) was used [26]. To estimate agreement on the non-metrical results, Fleiss’ Kappa coefficients were calculated. ICC and Fleiss’ Kappa coefficients were interpreted as shown in Table 1. For statistical analysis, SPSS 27 (IBM, Armonk, USA) and Prism 9.4 (GraphPad Software, California, USA) were used.

**Table 1:** Interpretations for Intra-Class Correlation and Fleiss’ Kappa coefficients

ICC	Interpretation^a^		Fleiss’ Kappa	Interpretation^b^
> 0.90	excellent		> 0.81	almost perfect
> 0.75	good		> 0.61	substantial
> 0.50	moderate		> 0.41	moderate
≤ 0.50	poor		> 0.21	fair

**a**: Koo & Li 2016, **b**: Landis & Koch, 1977

ICC = Intra-Class Correlation

An ordinal scale was created to evaluate treatment decisions by giving every non-operative treatment decision the number one and every operative treatment decision the number two. Thus a “mean treatment decision” ranging between one and two was calculated between all experts for every fracture. For mean values below 1.2, a consensus for non-operative treatment was concluded. Likewise, for mean values above 1.8, a consensus for operative treatment was supposed.

## Results

Twenty-two experts in pediatric traumatology assessed twenty anteroposterior (ap) radiographs of patients after a proximal humeral fracture. All images were assessed twice, blinded, and in random order using the online tool Tyche. Thus, a total of 880 assessments were made. The experts were asked to determine the fracture angulation and choose an operative or non-operative treatment. Apart from age, no patient history was provided.

The mean fracture angulation was 18.4° (SD = 15.4°). To estimate measurement accuracy, the mean of individual standard deviations between all observers was calculated as 8.5°, 46.2% in relation to the total mean of all measurements (18.4°). Based on Intra-Class Correlation (ICC) coefficients, interobserver reliability was moderate (0.6), while intraobserver reliability was excellent (0.9) (Table 2).

**Table 2:** Fracture angulation with standard deviation, accuracy and measurement reliability

Fracture angulation: Mean ± SD	18.4° ± 15.4
Mean of individual SDs (relative to total mean of 18.4°)	8.5° (46.2%)
Interobserver reliability: ICC_3,k_ [CI, *p*-value]	0.60 [0.47–0.75, 0.001]
Intraobserver reliability: ICC_3,1_ [CI, *p*-value]	0.90 [0.88–0.92, 0.001]

The studied population has a high SD and thus many different fracture angulations are represented. The mean of individual standard deviations was used to estimate measurement accuracy of individual measurements and is relatively high (46.2%). Using ICCs, interobserver reliability was moderate (0.6) while intraobserver reliability was excellent (0.9)

SD = Standard deviation, ICC = Intra-Class Correlation, CI = Confidence interval

The agreement on operative vs. non-operative treatment among experts was calculated using Fleiss’ Kappa. Interobserver reliability was fair (0.38), while intraobserver reliability was substantial (0.77) (Table 3).

**Table 3:** Low agreement between experts regarding the treatment

Interobserver reliability: Kappa [CI, *p*-value]	0.38 [0.36–0.40, 0.0001]
Intraobserver reliability: Kappa [CI, *p*-value]	0.77 [0.70–0.84, 0.0001]

Using Fleiss’ Kappa for treatment decisions (operative vs. non-operative), interobserver reliability was fair (0.38) while intraobserver reliability was substantial (0.77)

CI = Confidence interval

**Fig. 2:**
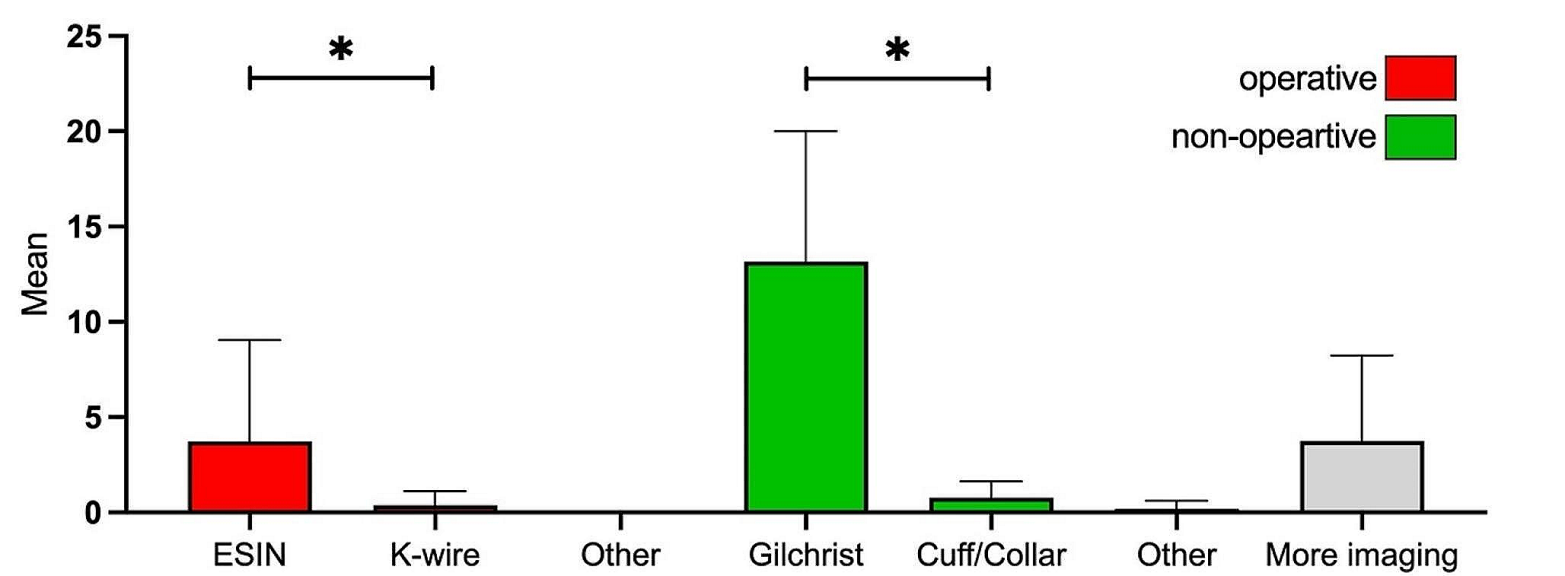
ESIN and Gilchrist are the preferred types of treatment For every fracture, experts were asked to choose one of seven treatment options (ESIN, K-wire, other operative; Gilchrist, Cuff/Collar, other non-operative; more imaging). The mean values across all images with standard deviations were calculated. Friedman test with multiple comparisons was used for statistical analysis; *p-values < 0.05

For 880 treatment decisions, 149 times (17%) ESIN was chosen for operative treatment. In total, 19% of fractures required surgery, according to the experts. A Gilchrist was chosen for non-operative treatment 527 times (60%). In total, 65% of fractures did not require surgery, as claimed by the experts. Other operative and non-operative treatments showed significantly fewer quantities (2% for K-wires and 4% for Cuff/Collar). 150 times (17%), more imaging was required, according to the experts (Table 4; Fig. 2).

**Table 4:** ESIN and Gilchrist are the preferred treatment modalities

Operative				Conservative				More imaging
ESIN	K-wire	Other		Gilchrist	Cuff/Collar	Other		
17% (149)	2% (15)	0		60% (527)	4% (31)	1% (8)		17% (150)

Twenty-two experts assessed twenty fractures twice. A total of 880 decisions were made. Experts were asked to choose the type of treatment or whether they needed more imaging to make a treatment decision. Most experts preferred ESIN for operative treatment and Gilchrist for non-operative treatment

**Fig. 3:**
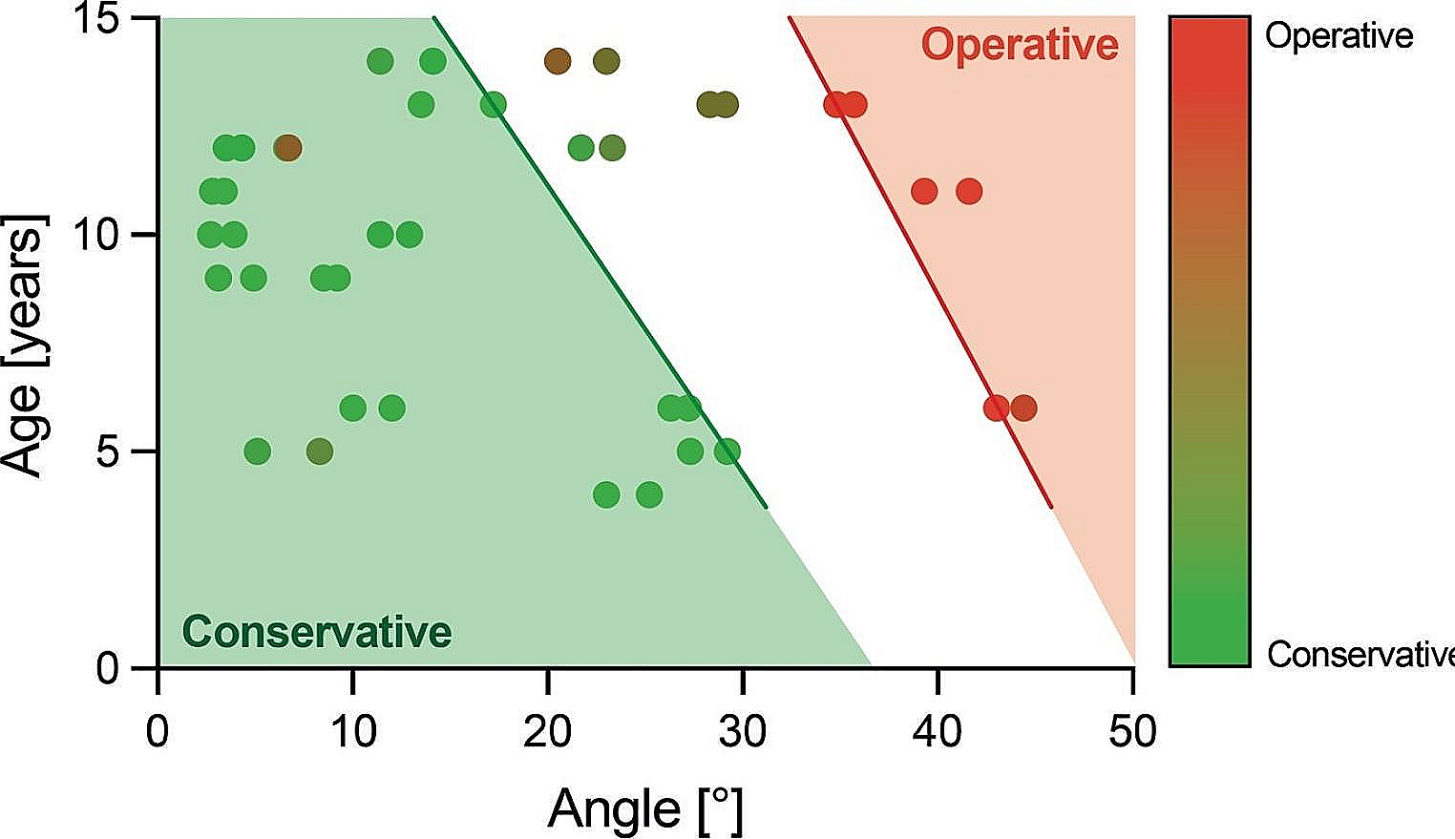
Recommended treatment based on fracture angulation and age Every dot represents one fracture, assessed by twenty-two experts. Dots are shown pairwise in proximity since every image was analyzed twice. An ordinal scale was created by giving the treatment decision “non-operative” the number 1 and the color green and giving “operative” the number 2 and red. A mean treatment among experts was calculated. The X-axis shows the mean fracture angulation, and the Y-axis the age. In the green area (left), most fractures were treated non-operatively (mean < 1.2). In the red zone (right), most fractures were treated operatively (mean > 1.8). One fracture lies in the green area despite the mean value above 1.2. Fracture dislocation other than angulation was not considered

The treatment decision based on age and fracture angulation is shown in Fig. 3. It shows that with an increase in age, less angulation was accepted for non-operative treatment by experts. Independent of age, fractures with angulation lower than 20° were usually chosen for non-operative treatment.

## Discussion

### Summary of results

The most important finding of this study was excellent intraobserver reliability (0.9) for the measurement of fracture angulation compared with only moderate interobserver reliability (0.6). Likewise, there was substantial intraobserver reliability (0.77) for the treatment decisions (operative vs. non-operative) compared to only fair interobserver reliability (0.38). In summary, for fracture angulations and treatment decisions, experts were consistent in their own assessments but very inconsistent with each other. High heterogeneity in treating PHFs in children in Germany can be concluded. Thus, there is a demand to standardize the measurement of fracture angulation and the treatment.

### Comparison to the literature

Burke et al. compared a new method for measuring fracture angulation with the observers’ standard method. Their new approach achieved excellent inter- and intraobserver reliability (0.96–0.97) in contrast to the observers’ traditional method achieving only moderate to excellent (0.74–0.84) reliability. In comparison, observers in our study achieved less interobserver (0.6) but higher intraobserver (0.9) reliability. This intraobserver reliability was achieved without specifying how angulation should be determined and included twenty-two experts from different locations. In contrast, Burke et al. included seven observers from one institution. Therefore, we conclude that a consensus on using one single measurement method is more important than a new or sophisticated one.

In our study, operative treatment was mostly recommended using ESIN. In contrast, according to the literature, percutaneous pinning using K-wires is the most common approach [4]. However, the German authors, like the experts questioned for this study, recommend using ESINs. For non-operative treatment, a Gilchrist was the preferred method. To our knowledge, there are no recommendations in the literature on whether one or the other is superior. There might be local differences according to availability.

### Limitations

This study has some limitations that need to be considered: performing X-rays on acutely injured children can be challenging and optimal ap view or angulation were not always guaranteed. Besides, fracture angulation may change before or after X-rays were acquired as immobilization is hard to accomplish, especially in young children. Hence, the absolute values reported, like the mean fracture angulation, need to be interpreted with caution. Moreover, treatment decisions should not solely rely on fracture angulation and age, as it is crucial to also take into account additional factors such as soft tissue damage, and vascular or nerve injuries. Nowadays, social factors like comfort, return to sports, less time of immobilization also play an important role. In summary, fracture angulation may never be the only factor guiding the treatment decision.

Additionally, the study was performed on only twenty images. Besides, the assessments were performed in only one session which could have allowed the experts to remember their first results, thus overestimating the intraobserver reliability. However, PHFs in children are rare, and twenty-two experts analyzed the fractures twice, which adds up to 880 assessments. Besides, the study population included a ratio of 3:1 for females to males, even though the fracture most commonly affects boys. The studied population might be less representative in terms of sex. However, sex plays a lesser role in treatment decisions than age and fracture angulation.

## Conclusion

Firstly, there is a need for consensus among experts on how fracture angulation in PHFs in children should be reliably determined. Our data indicate that choosing one method everybody agrees to use could be more helpful than using the most sophisticated. However, the overall importance of fracture angulation should also be critically discussed. Finally, experts should agree on treatment algorithms that could translate into guidelines to standardize the care and perform reliable outcome studies.

## Data Availability

No datasets were generated or analysed during the current study.

## Abbreviations

±: = Standard deviation
